# Psychomotor Function in Chronic Daily Cannabis Smokers during Sustained Abstinence

**DOI:** 10.1371/journal.pone.0053127

**Published:** 2013-01-02

**Authors:** Wendy M. Bosker, Erin L. Karschner, Dayong Lee, Robert S. Goodwin, Jussi Hirvonen, Robert B. Innis, Eef L. Theunissen, Kim P. C. Kuypers, Marilyn A. Huestis, Johannes G. Ramaekers

**Affiliations:** 1 Departments of Neuropsychology and Psychopharmacology, Maastricht University, Maastricht, The Netherlands; 2 Chemistry and Drug Metabolism, Intramural Research Program, National Institute on Drug Abuse, National Institute of Health, Baltimore, Maryland, United States of America; 3 Molecular Imaging Branch, National Institute of Mental Health, National Institute of Health, Bethesda, Maryland, United States of America; The Scripps Research Institute, United States of America

## Abstract

**Background:**

The present study assessed psychomotor function in chronic, daily cannabis smokers during 3 weeks continuously monitored abstinence on a secure research unit. We hypothesized that psychomotor performance would improve during abstinence of chronic, daily cannabis smokers.

**Methodology/Principal Findings:**

Performance on the critical tracking (CTT) and divided attention (DAT) tasks was assessed in 19 male chronic, daily cannabis smokers at baseline and after 8, 14–16 and 21–23 days of continuously monitored abstinence. Psychomotor performance was compared to a control group of non-intoxicated occasional drug users. Critical frequency (λ_c_) of the CTT and tracking error and control losses of the DAT were the primary outcome measures. Results showed that chronic cannabis smokers’ performance on the CTT (p<0.001) and the DAT (p<0.001) was impaired during baseline relative to the comparison group. Psychomotor performance in the chronic cannabis smokers improved over 3 weeks of abstinence, but did not recover to equivalent control group performance.

**Conclusions/Significance:**

Sustained cannabis abstinence moderately improved critical tracking and divided attention performance in chronic, daily cannabis smokers, but impairment was still observable compared to controls after 3 weeks of abstinence. Between group differences, however, need to be interpreted with caution as chronic smokers and controls were not matched for education, social economic status, life style and race.

## Introduction

Cannabis is the most commonly used illicit substance worldwide [1]. In 2009, approximately 1.7% of Americans 12 years or older were cannabis dependent [2], and greater than 1% of Europeans smoked cannabis daily or almost daily [3].

Long-term cannabis use is associated with neuropsychological deficits such as memory impairment (e.g. [4], [5], [6]) and changes in brain morphology [7]. Memory deficits appeared transient, as performance in long-term cannabis smokers returned to normal over 3 weeks of abstinence [8]. Preclinical and clinical research also indicated that alterations in endocannabinoids in the central nervous system after prolonged cannabis exposure might be transient. Chronic cannabis administration induced desensitization and CB_1_ receptor down-regulation in animals (e.g. [9], [10]), with receptor levels recovering after 1–2 weeks abstinence [10]. In humans, CB_1_ receptor density was down-regulated in chronic, daily cannabis smokers, returning to normal levels after 4 weeks abstinence [11]. Together, these data indicate that cannabis-related memory alterations and CB_1_ receptor down-regulation in chronic cannabis smokers are reversible and related to recent use, rather than irreversible and related to cumulative lifetime intake.It is not clear if other neuropsychological dysfunctions observed in long-term cannabis smokers such as psychomotor impairment, also are transient. Diminished brain activation in motor cortical circuits during a finger-tapping task 28 days after cannabis discontinuation [12] suggested motor impairments might persist during extended abstinence.

Δ^9^-tetrahydrocannabinol (THC) acute effects on psychomotor function are well known: stimulation of CB_1_ receptors by agonists including the endogenous ligand anandamide, induce deviant motor behaviors, such as catalepsy, immobility and ataxia (e.g. [13], [14]). In humans, single THC doses impaired motor control on neuropsychological tests measuring reaction time, tracking performance including actual driving tests, divided attention, and motor impulsivity [15], [16], [17], [18], [19], [20]. The present study assessed psychomotor function in chronic daily cannabis smokers during 3 weeks of sustained cannabis abstinence. Psychomotor function was assessed with critical tracking (CTT) and divided attention (DAT) tasks, and chronic cannabis smoker and occasional drug user performance was compared. This research was part of a larger clinical project exploring CB_1_ receptor availability during cannabis dependence and extended abstinence [11]. We expected chronic daily cannabis smoker baseline psychomotor function to be impaired relative to the comparison group, and for psychomotor function to improve over time during 3 weeks of abstinence.

## Subjects and Methods

### Ethics Statement

This study was conducted according to the code of ethics on human experimentation established by the declaration of Helsinki (1964) and amended in Seoul (2008). Approval for the study was obtained from the Institutional Review Board (IRB) of the National Institute of Mental Health (NIMH). All consent procedures used in this study were approved by the ethical committee.

### Participants

Nineteen chronic daily cannabis smokers, mean (SE) age 27.6 (1.5) years, participated in this sustained continuously monitored cannabis abstinence study. Participants self-reported smoking 10.9 (1.6) cannabis joints per day for the last 10.5 (1.2) years. Inclusion criteria were: male; 18–65 years of age; written informed consent; healthy, based on history and physical examination; smoked cannabis at least 5 days/week for 6 months prior to admission; and a positive urine cannabinoid test within 90 days. Exclusion criteria were: history or presence of any clinically significant illness; past or present diagnosis of schizophrenia, bipolar illness or any other psychotic disorder; need for psychoactive medication within preceding 28-days (42 days for fluoxetine); current physical dependence on any substance other than cannabis, nicotine or caffeine; positive HIV test; metallic foreign bodies or fear of enclosed spaces; head trauma resulting in a period of unconsciousness lasting longer than 10 minutes; history of fetal alcohol syndrome or other neurodevelopmental disorder; history of seizures; recent exposure to radiation; inability to lie flat on the camera bed for approximately 2.5 h; regular use of alcohol, i.e. ≥6 standard drinks per day four or more times per week in the month prior to study entry; currently interested in or participating in drug abuse treatment, or participation within 60 days preceding study enrollment.

The control group consisted of occasional cannabis and/or MDMA users. Control subjects performed the DAT (N = 30) and the CTT (N = 31) during placebo treatment as part of protocols to establish psychomotor function during MDMA [21], [22] or THC intoxication [17], [20] respectively. The latter studies were conducted at Maastricht University, The Netherlands and used identical test setting and procedures as compared to experimental group of chronic daily cannabis users. These studies were initially designed to assess effects of single MDMA and THC doses on psychomotor function in placebo controlled, cross-over studies. Placebo treatments served as the reference for drug-induced within subject changes. Wash-out period between treatments was at least 7 days in order to exclude cross-over effects of drug use during placebo treatment. Subjects tested negative for drugs in urine and blood prior to psychomotor testing during placebo. Their drug free, placebo performance was taken as a general reference level of performance for comparison to that of chronic daily cannabis smokers in the present study. Inclusion and exclusion criteria were identical for the control subjects as the abstinence group, except that control subjects included males and females and were subject to occasional drug use. Occasional drug use was defined as weekly use of cannabis or MDMA or less. Mean (SE) age of control subjects was 22.7 (0.3) years.

### Procedures and Study Design

Participants resided on the closed, secure Johns Hopkins Behavioral Pharmacology Research Unit, Baltimore, USA throughout the study. Performance tests were administered at NIDA and subjects were under continuous medical supervision. Psychomotor tests for measuring attention and motor performance included CTT and DAT administered on days 1 or 2 (baseline), day 8, day 14, 15 or 16, and day 21, 22 or 23. Psychomotor tests were developed and installed at the USA testing site by research team members from Maastricht University, The Netherlands. Psychomotor data obtained with CTT and DAT can be very susceptible to practice effects and usually shows a learning curve over task repetitions. As a consequence, subjects need to receive extensive training to achieve a stable and reliable performance level and to exclude practice effects prior to study participation. A standard training protocol for the CTT and DAT was used for the experimental and control group in the present study. Basically, all subjects received extensive training on the psychomotor tests (at least 20 repetitions) until stable performance was achieved (less than 10% variance in 5 consecutive measurements) prior to baseline (experimental group) or placebo (controls). The same training protocol has been successfully applied in a range of alternate double-blind, placebo controlled studies (e.g. [23], [24], [25]) showing that well trained subjects achieve stable psychomotor performance levels under repeated testing during 1–2 weeks of placebo treatment.

### Critical Tracking Task

The CTT measures the subject’s ability to control a displayed error signal in a 1st-order compensatory tracking task. Error appears as horizontal deviation of the cursor from midpoint on a horizontal, linear scale. Compensatory joystick movements null the error by returning the cursor to the midpoint. The frequency of cursor deviations, and therefore its velocity, increases as a stochastic, linear function of time. The subject is required to make compensatory movements with a progressively higher frequency until the subject is unable to correct the deviation. The frequency at which control loss occurs is λ_c_ (the critical frequency). The reciprocal of this frequency is theoretically the perceptual/motor delay lag for humans operating in a closed-loop system. The participant performs this test in five trials and the mean λ_c_ is recorded as the final score [26]. The test has demonstrated sensitivity to the impairing effects of THC [16], [17], [20].

### Divided Attention Task

The DAT assesses the ability to divide attention between two tasks performed simultaneously. The primary task required the use of a joystick to continuously null the horizontal movement of a cursor from the center of a display as described above in the CTT. The velocity of the cursor was kept constant at 50% of the participant’s optimal performance (λ_c_/2). The dependent measure of this subtask was control losses, i.e. the number of times a participant could not keep the cursor within a predefined range. Tracking error, measured by the absolute distance (mm) between the cursor's position and the center was another dependent measure of this subtask. The secondary task involved monitoring 24 single-digit numbers (0–9) arranged in the four corners of the display. The numbers changed asynchronously every 5 seconds. The requirement was to react as rapidly as possible by lifting the foot from a pedal every time a target, i.e. the number 2, appeared. Average reaction time to target and percentages hit were recorded as the dependent measure [27]. The DAT also demonstrated sensitivity to THC’s impairing effects [16], [20].

### Pharmacokinetic Assessment

Oral fluid and blood were collected before each test session, i.e. on days 1 or 2 (baseline), 8, 14–16 and 21–23. Blood was centrifuged, plasma separated and samples frozen at −20°C until analysis. Plasma specimens were analyzed for THC, 11-OH-THC and THCCOOH concentrations by a previously published analytical method [28]. Oral fluid samples were collected with the Immunalysis Quantisal™ device, with a volume adequacy indicator. The pad, placed into the participant’s mouth until the designated volume (1.0±0.1 mL) was collected and subsequently placed into a plastic tube containing 3 mL elution and stabilizing buffer, yielding a 1∶4 oral fluid dilution. The tube was capped and refrigerated for at least 24 h. Pads were squeezed dry with a serum separator before decanting into a Nunc® cryotube and stored at −20°C before analysis. Oral fluid samples were analyzed for THC, CBD, CBN, and THCCOOH according to a previously published 2-dimensional gas chromatography-mass spectrometry (2D-GC-MS) method employing two analytical systems with different ionization techniques [29].

### Statistical Analysis

All statistical analyses were performed with SPSS 19.0 for Mac. Between (chronic smokers versus controls) and within group comparisons (days of abstinence) were conducted in two separate analyses, since the within group factor was present in the experimental group only. A general linear model (GLM) repeated measures analysis of variance (ANOVA) was performed on data from chronic cannabis smokers with days of abstinence (3 levels: baseline, day 8, day 14–16) as the main within subjects factor to assess overall differences in performance over the test days. If the sphericity assumption was violated, the Greenhouse-Geisser correction was used. Assessments of overall effects of days of abstinence were followed by simple contrasts comparing performance on day 8 and 14–16 versus baseline. Data collected on days 21–23 were not included in the overall analyses but contrasted separately to baseline, because multiple subjects withdrew from the study prior to 3 weeks abstinence. The association between task performance and withdrawal effects was assessed using Pearson r correlations. Differences between control and experimental groups at baseline and during abstinence were tested using a one-way ANOVA with planned comparisons. Associations between gender, age and task performance were assessed to determine the clinical relevance of existing gender and age differences between groups. Gender effects on task performance were determined in the control group by means of T-tests, whereas the association between age and task performance was assessed by means of Pearson-r correlations in both groups.

### Clinical Trial Registration

Clinicaltrials.gov NCT00816439.

## Results

### Missing Values

DAT data sets were complete for 19 subjects at baseline and on days 14–16. Data from one subject was missing on day 8. CTT data from 2 subjects (one on day 8 and one on day 14–16) were missing. In total, data from 17 subjects were available for the CTT repeated measures analysis. By day 21–23, seven subjects voluntarily withdrew from the study for family reasons or because they no longer wanted to remain on the closed research unit.

### Effects of Gender and Age

Performance on the CTT and DAT did not significantly differ between males and females in the control group as assessed by a T-test (t(29) = 0.021, p = 0.983; t(28) = −0.829, p = 0.414; t(28) = −0.770, p = 0.448 for the CTT, control losses and tracking error of the DAT respectively). Mean age of chronic daily smokers significantly (t(19.9) = 3.13, p = 0.005) differed from mean age of the control group by about 5 years (28 versus 23 years). Pearson R analyses however revealed that age was not significantly correlated with critical tracking and divided attention performance parameters of chronic daily smokers (at baseline) and controls (r = −0.06, p = 0.679; r = −0.049, p = 0.738; r = 0.276, p = 0.055 for the CTT, control losses and tracking error of the DAT respectively). Table 1 shows mean (SE) performance on the CTT and DAT of controls and chronic, daily cannabis smokers at baseline, and after 8, 14–16, and 21–23 days of abstinence.

**Table 1:** Mean (SE) critical tracking task and divided attention performance in chronic, daily cannabis smokers and controls.

Cannabis use history	Chronic cannabis smokers				Controls
Days of abstinence	Baseline	Day 8	Day14–16	Day21–23	
Critical tracking task	N = 19	N = 18	N = 19	N = 12	N = 30
λ_c_ (rad/s)	2.8 (0.2)	3.1 (0.2)	3.1 (0.1)	3.0 (0.3)	3.9 (0.1)
Divided attention task	N = 19	N = 18	N = 18	N = 12	N = 30
Control loss (N)	23.4 (8.2)	8.7 (3.1)	5.6 (1.6)	8.8 (2.1)	1.2 (0.4)
Tracking error (mm)	19 (1.0)	18 (1.2)	17 (1.2)	17 (1.8)	14 (0.8)
Hit (%)	87.2 (2.8)	89.5 (2.6)	87.0 (4.4)	85.4 (5.7)	94.6 (1.3)
RT (ms)	1892 (72)	1828 (70)	1854 (101)	1830 (74)	1880 (53)

### Critical Tracking Task

**Figure 1:**
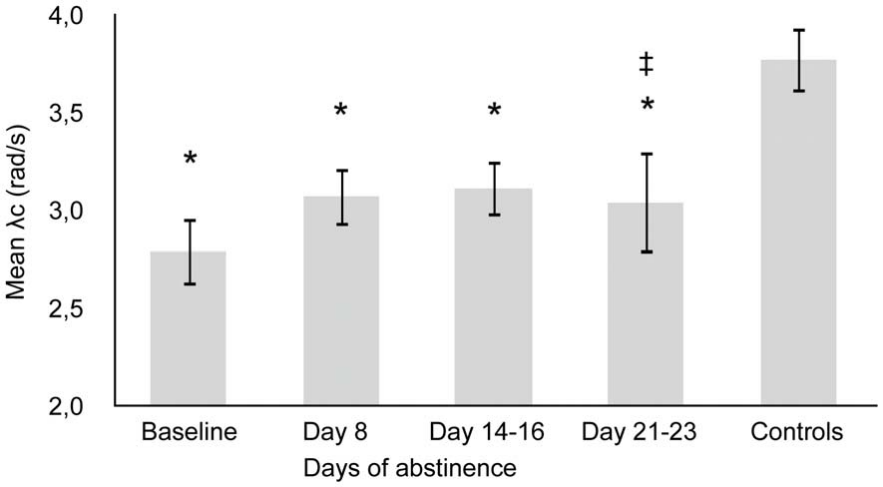
Mean (SE) λ_c_ in the critical tracking task as a function of time of abstinence in chronic daily cannabis smokers. ‡ indicates significant difference (p<0.05) from baseline; * indicates significant difference (p<0.05) from control group. N = 19 at baseline, N = 18 on day 8, N = 19 on days 14–16, N = 12 on days 21–23 and N = 30 for controls.

Mean λ_c_ demonstrated a trend effect of Days of abstinence (F_2,32_ = 2.540, p = 0.095). Simple contrasts indicated that subjects performed better after 8 days (F_1,16_ = 3.203, p = 0.092), after 14–16 days (F_1,16_ = 3.487, p = 0.080) and after 21–23 days of abstinence (F_1,11_ = −5.096, p = 0.045) compared to baseline. Age of first cannabis use was significantly correlated with CTT performance at baseline (r = 0.688, p = 0.001) and after 14–16 days of abstinence (r = 0.605, p = 0.008). After 14–16 days of abstinence CTT performance was significantly correlated with withdrawal effects (r = −0.479, p = 0.044). At baseline and after 8 days of abstinence the correlation approached significance (r = −0.439, p = 0.060 and r = −0.423, p = 0.081 respectively). CTT performance of chronic, daily smokers also differed significantly from that of the control group (F_4,93_ = 8.629, p<0.001). Planned comparisons noted differences at baseline (p<0.001), after 8 (p = 0.002), 14–16 (p = 0.004) and 21–23 days of abstinence (p = 0.008) compared to controls. Mean (SE) performance is shown in Figure 1.

### Divided Attention Task

**Figure 2:**
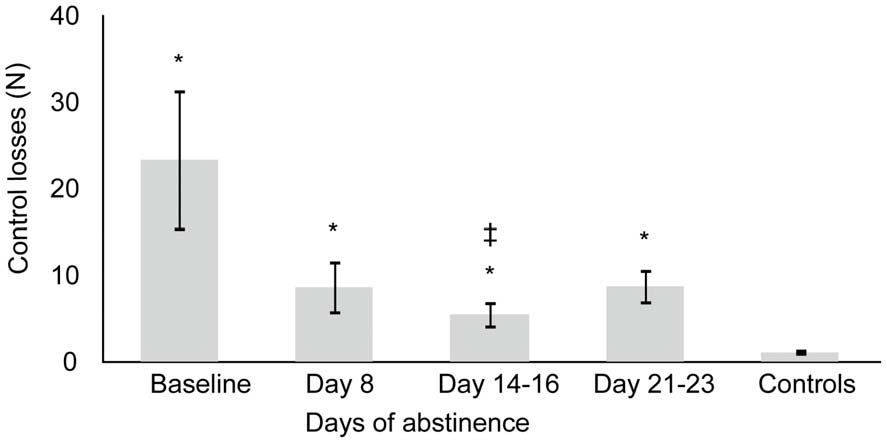
Mean (SE) number of control losses in the divided attention task as a function of time of abstinence in chronic daily cannabis smokers. ‡ indicates significant difference (p<0.05) from baseline; * indicates significant difference (p<0.05) from control group. N = 19 at baseline, N = 18 on day 8, N = 19 on days 14–16, N = 12 on days 21–23 and N = 30 for controls.

Overall, the number of control losses decreased during abstinence and approached statistical significance (F_1.186,20.160_ = 3.495, p = 0.070). Simple contrasts showed that the number of control losses significantly decreased after 14–16 days of abstinence (F_1,17_ = 4.611, p = 0.046), but trended at 21–23 days of abstinence (F_1,11_ = 3.850, p = 0.076), relative to baseline. In addition, linear contrasts suggested that the number of control losses decreased in a linear manner during 2 weeks of abstinence. (F_1,17_ = 4.611, p = 0.046). Compared to the control group, chronic, daily cannabis smokers had significantly more control losses (F_4,93_ = 5.051, p = 0.001). Planned comparisons indicated this was true at baseline (p = 0.014), 8 (p = 0.029), 14–16 (p = 0.017) and 21–23 days of abstinence (p = 0.004). Mean (SE) number of control losses in both groups are displayed in Figure 2.

**Figure 3:**
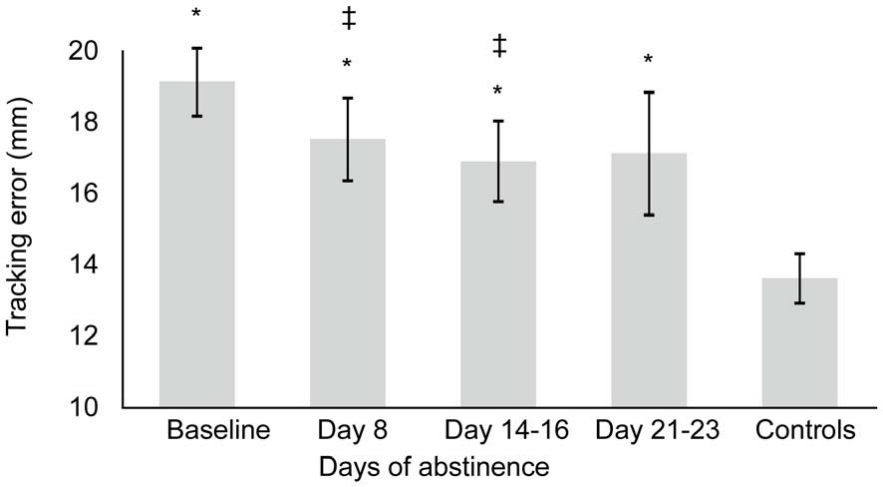
Mean (SE) tracking error on the divided attention task as a function of time of abstinence in chronic daily cannabis smokers. ‡ indicates significant difference (p<0.05) from baseline; * indicates significant difference (p<0.05) from control group. N = 19 at baseline, N = 18 on day 8, N = 19 on days 14–16, N = 12 on days 21–23 and N = 30 for controls.

Tracking error decreased during abstinence (F_2,34_ = 7.226, p = 0.002) in a linear manner (F_1,17_ = 13.370, p = 0.002) over 2 weeks of abstinence. Simple contrasts indicated that tracking error of daily cannabis smokers significantly decreased after 8 (F_1,17_ = 5.382, p = 0.033) and 14–16 days of abstinence (F_1,17_ = 13.370, p = 0.002). The decrease in tracking error after 21–23 days of abstinence approached significance (F_1,11_ = 4.028, p = 0.070). Age of first cannabis use was significantly correlated with tracking error after 21–23 days of abstinence (r = −0.754, p = 0.005). Control losses were significantly related to withdrawal effects at baseline (r = 0.693, p = 0.001) and after 14–16 days of abstinence (r = 0.840, p<0.001). Tracking error was not correlated with withdrawal symptoms. Compared to controls, chronic daily cannabis smokers displayed significantly larger tracking error (F_4,93_ = 4.315, p = 0.003). Planned comparisons showed significant increments in tracking error of chronic cannabis smokers at baseline (p<0.001), after 8 (p = 0.008), 14–16 (p = 0.024) and 21–23 days of abstinence (p = 0.037) compared to controls. Mean (SE) tracking error in both groups is displayed in Figure 3.

Reaction time and percentages hits of the experimental group did not differ during abstinence or in comparison to the control group.

### Pharmacokinetic Assessment

Mean THC, 11-OH-THC and THCCOOH plasma concentrations and THC and THCCOOH oral fluid concentrations are shown in Table 2. At baseline, 1 subject (5.3%) was negative for THC in plasma, although positive for THCCOOH; negative THC results increased to 15.8% of subjects after 8 and 14–16 days of abstinence. After 21–23 days of abstinence, 33.3% of subjects were plasma THC negative.

**Table 2:** Mean (SE) THC, 11-OH-THC and THCCOOH concentrations and percentage THC negative subjects in plasma and THC and THCCOOH concentrations in oral fluid of chronic daily cannabis smokers as a function of days of abstinence.

	Plasma				Oral fluid	
Days of abstinence	% THC negative	THC(µg/L)	11-OH-THC(µg/L)	THCCOOH(µg/L)	THC(µg/L)	THCCOOH(ng/L)
Baseline (N = 19)	5.3	5.3 (1.2)	2.1 (0.4)	54.2 (9.0)	29.5 (11.5)	48.2 (8.4)
Day 8 (N = 19)	15.8	1.3 (0.2)	0.2 (0.1)	6.9 (1.0)	0.0 (0.0)	4.4 (2.8)
Day 14–16 (N = 19)	15.8	0.8 (0.2)	0.1 (0.04)	4.0 (0.6)	0.0 (0.0)	5.1 (2.8)
Day 21–23 (N = 12)	33.3	0.4 (0.2)	0.0 (0.0)	2.2 (0.7)	0.0 (0.0)	2.5 (2.5)

## Discussion

This study was designed to assess the effects of cannabis abstinence on psychomotor performance in chronic, daily cannabis smokers. Their performance on the CTT and DAT was compared to an occasional drug-using control group. Results showed that psychomotor performance of chronic, daily cannabis smokers improved throughout 3 weeks of abstinence, but remained significantly poorer than performance of a control group of occasional drug users. Overall, mean performance changes from baseline appeared more prominent during weeks 1 and 2 of abstinence, and less so in week 3. This apparent inconsistency may, however, be related to the reduction in sample size during the last phase of the study.

The CTT measures perceptual motor control. In essence, the task assesses human operator performance when the person perceives a discrepancy between a desired and actual state and aims to reduce the error by compensatory movement during a continuous closed-loop system. Chronic, daily cannabis smokers performed relatively poorly on this task, indicating that they were slow in initiating a compensatory response to error signals when compared to controls. Mean CTT performance significantly improved after 3 weeks of abstinence compared to baseline, demonstrating recovery of critical tracking abilities in chronic cannabis smokers. However, critical tracking performance did not fully recover after 3 weeks of abstinence and was still significantly worse compared to critical tracking in the control group. Similar results were obtained in the DAT. At baseline, tracking performance and tracking control were impaired in chronic, daily cannabis smokers compared to the control group; i.e. tracking error and number of control losses were significantly higher in the chronic smokers. During 2 weeks of abstinence, tracking performance and control significantly improved, but remained impaired as compared to controls. Due to a loss of statistical power in the 3rd week, no significant improvement was demonstrated at this time compared to baseline. Together, these results indicate prolonged impairment of psychomotor function in chronic cannabis smokers that only partially recovered over 3 weeks of continuously monitored abstinence.

In the past, several explanations were suggested to account for impairments observed in chronic cannabis smokers. Some proposed that such impairments arose from withdrawal from daily cannabis use [30].The present data partly confirmed this notion as withdrawal contributed to the number of control errors in the DAT. However tracking performance in the DAT and the CTT were not or only weakly related to withdrawal.

It was also suggested that impairments in chronic cannabis smokers may result from residual THC concentrations in blood [8], which may remain present in sustainable amounts for several days after last use [20], [31], [32]. This finding is also illustrated by the fact that in the present study, 8 of 12 chronic smokers were still positive for THC after 3 weeks of abstinence. However, mean THC concentrations were below concentrations (i.e. 2–5 µg/L in serum), showing THC psychomotor impairment [17], [20]. Residual THC concentrations, therefore, may not account for the psychomotor impairment observed in chronic cannabis smokers in the present study.

Alternatively, prolonged impairment may have resulted from cumulative lifetime intake and reflect persistent changes in psychomotor functions in chronic cannabis smokers. Previous reports indicated that recently abstinent smokers had reduced activation in motor cortical areas and that such deactivation could still be observed after 28 days of cannabis discontinuation [12]. Moreover, subjects in the present study started their cannabis use early in life (mean 14.7 years), and their age of first cannabis use was negatively correlated with magnitude of performance impairment. This fits with previous research suggesting that cannabis use at an early age is a risk factor for developing long-term neuropsychological dysfunction in chronic cannabis smokers [33]. We also demonstrated that divided attention performance increased linearly during 2 weeks of abstinence, suggesting that psychomotor function may continue to improve over time. Based on our data, it is not possible to predict when or if psychomotor function in chronic cannabis smokers would return to performance demonstrated in occasional cannabis smokers. Additional data following longer periods of abstinence (i.e. 6–12 months) are needed, although documentation of sustained abstinence in a naturalistic setting would be difficult. A strength of the current study was residence on a secure research unit with continuous monitoring for more than 3 weeks, precluding cannabis relapse.

The neurobiological mechanism underlying (partial) recovery of psychomotor function during abstinence is unknown, but might be related to increments in CB_1_ receptor density observed in these chronic cannabis smokers after 4 weeks of abstinence [11]. Positron emission tomography documented CB_1_ receptors down-regulation at baseline but increased receptor density in cortical brain regions after sustained abstinence. Down-regulation was not demonstrated in subcortical regions, such as basal ganglia, midbrain and cerebellum. Cortical regions, such as the prefrontal cortex, are important for cognitive processing, an integral component of most psychomotor tasks, including CTT and DAT. Alternatively, it cannot be fully excluded that a change in lifestyle factors accompanying residence on a closed research unit (i.e. regular sleep and/or abstinence of alcohol) also contributed to the improvement in psychomotor performance.

Some potential limitations of the current study should be noted. Chronic daily cannabis smokers (USA) were not fully matched with controls from Dutch occasional drug users. Groups slightly differed in mean age (i.e. 23 vs. 28 years) and also in gender, race and education. Chronic cannabis smokers were males, whereas controls were males and females. The Dutch sample were Caucasian, whereas the USA sample were African-American and Caucasian. Correlation analysis revealed that psychomotor performance of subjects in both groups was not significantly correlated with age indicating that age differences did not affect psychomotor function. Likewise, psychomotor performance did not significantly differ between males and females in the control group. The latter implies that gender differences between chronic cannabis smokers and controls did not contribute to observed performance differences. The relevance of race for divided attention and tracking performance is presently unknown and could not be determined in the current sample. Other factors that may have contributed to performance impairment in chronic cannabis smokers were polydrug use (including alcohol) and differences in health and lifestyle or socioeconomic status between chronic and occasional drug users.

It might also be argued that the present study should have utilized a different control group, such as chronic cannabis smokers or non-drug users. There also are good arguments for occasional drug users, as employed here. The times of last cannabis smoking would be difficult to determine in chronic cannabis smokers, as would ensuring a lack of intoxication. Comparisons between healthy, non-drug users and drug users may also be biased since differences in neuropsychological function may have already pre-existed in drug users and prompted their drug use. We aimed to avoid these potential biases by selecting a control group that also had a history of (occasional) drug use, but was negative for any drugs at time of testing.

Finally, the control group and the experimental group were not controlled for time on task effects. Psychomotor performance in abstinent smokers was repeatedly assessed over a 3 week period, whereas the control group’s performance was assessed at a single time point. This raises the possibility that performance improvement observed in abstinent users resulted from practice effects rather than a recovery during abstinence. However, it should be noted that participants in the experimental and control group were optimally trained to achieve stable performance levels before they entered the actual study phase (see section procedures and study design for more details). These standard training protocols have been applied in previous studies and were demonstrated to bring subjects to stable performance levels under repeated testing during 1–2 weeks of placebo treatments (e.g. [23], [24], [25]). We therefore believe that the influence of practice effects during repeated testing were minimal or absent in the present study since these were already controlled for during the training phase.

In sum, psychomotor function of chronic cannabis smokers improved during 3 weeks of monitored abstinence, but did not recover to a normal performance level as assessed in a control group of occasional drug users. Between group differences however need to be interpreted with caution since chronic smokers and controls were not matched for education, social economic status, life style and race.
